# Risk prediction model for pegaspargase-associated severe coagulopathy and blood product costs: a single-center retrospective cohort study

**DOI:** 10.3389/fphar.2026.1893967

**Published:** 2026-10-07

**Authors:** Xiangyu Ding, Zhonghao Xu, Chenhong Jia, Sitong Wang, Deyun Zhao

**Affiliations:** 1 Department of Pharmacy, Hebei Children’s Hospital, Shijiazhuang, Hebei, China; 2 Hebei Provincial Clinical Research Center for Child Health and Disease, Shijiazhuang, Hebei, China; 3 Department of Personnel and Organization, Hebei Children's Hospital, Shijiazhuang, Hebei, China; 4 College of Pharmacy, Hebei Medical University, Shijiazhuang, Hebei, China

**Keywords:** acute lymphoblastic leukemia, blood products, nomogram, pediatrics, pegaspargase, risk prediction model, severe coagulopathy

## Abstract

**Introduction:**

Pegaspargase-induced severe coagulopathy (SC) is a common complication in pediatric acute lymphoblastic leukemia (ALL) treatment, yet reliable predictive tools are limited and its economic burden remains unclear. This study developed and internally validated a clinical prediction nomogram for SC, concurrently created a web-based calculator, and quantified the impact of coagulopathy on the cost of blood products.

**Methods:**

Using retrospective data from 356 pediatric patients treated according to the Chinese Children’s Leukemia Group Acute Lymphoblastic Leukemia 2018 protocol at Hebei Children’s Hospital between January 2020 and August 2025, SC was defined as grade 4 fibrinogen reduction or grade ≥3 prolongation of the activated partial thromboplastin time. A nomogram was developed via multivariate logistic regression, and cost drivers were analyzed by multiple linear regression.

**Results:**

Hypoalbuminemia, elevated bilirubin, and elevated procalcitonin were independent predictors of SC. The nomogram achieved an area under the curve of 0.819 (95% CI: 0.773–0.864), and decision curve analysis indicated favorable clinical utility. Blood product cost was primarily driven by patient age, product type, and transfusion frequency (adjusted *R* ^2^ = 0.677).

**Discussion:**

This study developed an accurate predictive model for SC and clarified the associated economic burden, providing evidence to support clinical vigilance and inform healthcare resource planning.

## 1 Introduction

Acute lymphoblastic leukemia (ALL) is the most common childhood malignancy. The incorporation of pegaspargase into combination chemotherapy regimens has contributed to an overall survival rate of 90% (Lynggaard et al., 2023). In clinical practice, pegaspargase is associated with multiple adverse reactions, among which coagulation abnormalities are commonly observed. Coagulation abnormalities related to pegaspargase use may present as both thrombosis and bleeding. Severe coagulopathy (SC) can necessitate chemotherapy interruption, potentially diminishing treatment efficacy and increasing healthcare costs (Torere et al., 2023). According to the Chinese expert consensus, the incidence of PEG-asparaginase-related coagulation and fibrinolysis abnormalities is approximately 25%–60% (Chinese Society of Clinical Oncology Leukemia and Lymphoma Study Group of Hematology BranchChinese Medical Association Pediatric Hematology BranchChinese Medical Association and United China Lymphoma Alliance, 2015). However, SC can often be managed through early intervention, thereby facilitating continuation of therapy (Bender et al., 2021). Accordingly, early identification of children at high risk of SC is clinically important for implementing risk-stratified management strategies and preventive measures.

A major challenge is that abnormalities detected by routine *in vitro* coagulation tests do not reliably reflect *in vivo* hemostatic function (National Health Commission of the People’s Republic of China, 2018). This discrepancy underscores the challenge of relying solely on laboratory parameters for clinical risk assessment. Although pegaspargase-associated coagulopathies are widely recognized clinically, simple and effective tools for predicting individual risk of SC in children are lacking. Most existing studies have focused on descriptive statistics or univariate analyses (Li et al., 2017; Xu et al., 2016; Zhang et al., 2018) but failed to integrate multiple indicators into a quantifiable and visual predictive model to support rapid and accurate risk assessment by clinicians.

Once coagulation disorders are established, clinical management often requires transfusion of blood products, such as fresh frozen plasma and cryoprecipitate, which increases treatment complexity and associated medical costs. To date, the specific impact of coagulation disorders on blood product usage and related expenditures has not been systematically evaluated.

In this single-center retrospective cohort study, we identified independent risk factors for pegaspargase-associated SC, developed a clinically useful risk nomogram, and assessed its clinical utility. We also conducted a quantitative evaluation of factors affecting blood product costs. Our objective was to enable early detection of children at high risk of SC, enhance treatment safety and continuity, and provide empirical evidence to guide clinical management and resource utilization.

## 2 Materials and methods

### 2.1 Study population

This retrospective study included children initially diagnosed with ALL at Hebei Children’s Hospital between January 2020 and August 2025. All participants received standardized treatment according to the Chinese Children’s Leukemia Group Acute Lymphoblastic Leukemia 2018 (CCLG–ALL–2018) protocol. Patients were grouped into the SC (case) and non-SC (control) groups based on whether they experienced SC during pegaspargase treatment.

The study protocol was approved by the Ethics Committee of the Hebei Children’s Hospital (Ethical Review Number: 202407–63). Given the study’s retrospective design and use of anonymized data, informed consent was not required.

### 2.2 Inclusion criteria

1. Diagnosed with ALL at Hebei Children’s Hospital between January 2020 and August 2025 and treated according to the CCLG-ALL-2018 protocol (National Health Commission of the People’s Republic of China, 2018).
2. Definitive diagnosis established through diagnostic evaluation and classification based on morphology, immunophenotype, cytogenetics, and molecular biology.
3. According to the classification of coagulation disorders in the Common Terminology Criteria for Adverse Events, Version 5.0 (CTCAE 5.0; National Cancer Institute, 2017), published by the National Cancer Institute of the United States (National Cancer Institute, 2017) Common Terminology Criteria for Adverse Events (CTCAE) Version 5.0. NIH (2025), this study defined SC as a fibrinogen level reduction of grade 4 or an activated partial thromboplastin time (APTT) prolongation of grade ≥3.

### 2.3 Exclusion criteria

1. Missing baseline or key clinical data during treatment.
2. SC occurring before the first pegaspargase exposure.
3. Failure to complete chemotherapy protocol with pegaspargase in CCLG-ALL-2018 (phases: intensive induction, remission consolidation, and delayed intensification; continuation treatment not included).

### 2.4 Data collection

Clinical data were systematically collected for all eligible patients, with each patient treated as an independent study unit. The collected variables were classified into three categories.

1. Clinicodemographic characteristics: age, sex, body surface area, immunophenotype, and disease risk stratification.
2. Treatment-related information: timing of coagulopathy onset, treatment regimen, and types and costs of blood products administered.
3. Laboratory indicators: platelet count (PLT), albumin, transaminases, bilirubin, C-reactive protein (CRP), and procalcitonin (PCT).

Considering that children may be exposed to pegaspargase multiple times, potentially leading to recurrent coagulation abnormalities, we strictly defined the index event for each patient. In the SC group, cases were recorded at the first occurrence of grade 4 fibrinogen reduction or grade ≥3 APTT prolongation. In the non-SC group, the index time point was defined as the first occurrence of the lowest fibrinogen level or the highest APTT value during follow-up.

To minimize the influence of temporal fluctuations in laboratory parameters during chemotherapy, all on-treatment laboratory indicators were assessed within a predefined window extending from 1 week before to 1 week after the nadir of fibrinogen following pegaspargase exposure. For each analyte, the extreme values within this window (minimum or maximum, as clinically appropriate) were extracted for analysis.

### 2.5 Statistical methods

All statistical analyses and figures were generated using R version 4.5.2 (R Foundation for Statistical Computing, Vienna, Austria), with extension packages including “rms”, “riskRegression”, “pROC”, “Hmisc”, “glmnet”, “nortest”, “dplyr”, “rmda”, “ggplot2”, and “forestplot”. Statistical significance was set at a two-sided *P* < 0.05.

The normality of continuous variables was assessed before analysis. Normally distributed data are presented as mean ± standard deviation (x̄ ± s) and were compared using the Student’s t-test. Nonnormally distributed variables are expressed as median and interquartile range (IQR) and were analyzed using the Mann–Whitney U test (or the Wilcoxon rank-sum test for paired samples, if applicable). Categorical variables are summarized as frequency (percentage) and were compared using the chi-square test (or Fisher’s exact test when expected cell counts were <5).

Variable selection was performed using stepwise regression. Multivariate logistic regression was applied to develop a predictive model for SC, followed by nomogram generation. Internal validation involved 1,000 bootstrap resamples. Model discrimination was assessed using the receiver operating characteristic (ROC) curve and the area under the curve (AUC). Calibration curves were used to assess agreement between predicted outcomes and actual incidence. Decision curve analysis (DCA) was used to measure the model’s clinical utility. Additionally, multiple linear regression was used to examine factors affecting blood product costs in pediatric patients. The assumptions of the regression model, including independence of residuals and absence of multicollinearity, were assessed. The Durbin–Watson (DW) statistic was calculated to test for residual autocorrelation, and variance inflation factors (VIF) were computed to detect multicollinearity among predictor variables.

## 3 Results

### 3.1 Basic characteristics of the study population

Between January 2020 and August 2025, 374 patients were diagnosed with ALL. Eighteen patients were excluded based on the predefined criteria: 10 had pre-existing coagulopathy prior to pegaspargase exposure, 2 discontinued pegaspargase due to severe pancreatitis, and 6 died during treatment (1 from cerebral venous sinus thrombosis and 5 from infection). The remaining 356 patients were included in the final analysis. Of these, 199 (55.90%) were male, and 157 (44.10%) were female, with a median age of 5.0 years. Most patients (89.89%) had B cell ALL, and 69.10% were classified as intermediate risk. Baseline characteristics between the SC group and non-SC group are listed in Table 1.

**TABLE 1:** Baseline data and characteristics (n = 356).

Characteristic	Overall (*n* = 356)	Non-SC (*n* = 218)	SC (*n* = 138)
Age (years)	5.0 (3.4, 7.8)	4.6 (3.2, 7.1)	5.8 (3.9, 8.7)
Body surface area (m^2^)	0.73 (0.61, 0.96)	0.70 (0.60, 0.90)	0.75 (0.66, 1.00)
Sex	​	​	​
Male	199 (55.90)	126 (57.80)	73 (52.90)
Female	157 (44.10)	92 (42.20)	65 (47.10)
Immunophenotyping			
B-ALL	320 (89.89)	197 (90.37)	123 (89.13)
T-ALL	36 (10.11)	21 (9.63)	15 (10.87)
Risk group			
LR	52 (14.61)	32 (14.68)	20 (14.49)
IR	246 (69.10)	144 (66.06)	102 (73.91)
HR	58 (16.29)	42 (19.27)	16 (11.59)
Laboratory findings			
WBC (×10^9^/L)	9.0 (4.0, 24.0)	9.0 (4.0, 23.0)	9.0 (4.0, 29.0)
<50	297 (83.43)	182 (83.49)	115 (83.33)
50–299	51 (14.33)	31 (14.22)	20 (14.49)
≥300	8 (2.25)	5 (2.29)	3 (2.17)
APTT (s)	26.8 (24.7, 29.3)	26.8 (24.5, 29.3)	26.8 (24.8, 29.4)
Fibrinogen (g/L)	2.97 (2.25, 3.83)	3.07 (2.39, 3.92)	2.81 (2.12, 3.55)
D-dimer (mg/L)	0.91 (0.47, 2.29)	0.96 (0.50, 2.54)	0.81 (0.45, 1.75)
Albumin (g/L)	41.1 (38.6, 43.8)	41.0 (38.2, 43.6)	41.2 (39.2, 43.9)
ALT (U/L)	14 (9, 23)	13 (9, 21)	14 (9, 29)
AST (U/L)	28 (21, 43)	28 (21, 42)	29 (20, 43)
TBIL (μmol/L)	5.6 (4.0, 8.9)	5.7 (3.9, 8.9)	5.5 (4.3, 8.9)

Abbreviations: B-ALL, B cell acute lymphoblastic leukemia; T-ALL, T cell acute lymphoblastic leukemia; LR, low risk; IR, intermediate risk; HR, high risk; WBC, white blood cell; APTT, activated partial thromboplastin time; ALT, alanine aminotransferase; AST, aspartate aminotransferase; TBIL, total bilirubin; IQR, interquartile range.

All quantitative data are presented as median (IQR), and categorical data are expressed as n (%).

Of 356 patients, 138 (38.76%) developed SC (Table 2). Within this subgroup, 93 exhibited grade 4 fibrinogen reduction, 17 demonstrated grade ≥3 APTT prolongation, and 28 presented with both abnormalities. Abnormalities in coagulation parameters predominantly occurred during vincristine, daunorubicin, L-asparaginase, and prednisone/dexamethasone (VDLP/VDLD) treatment regimens. The median interval from the first pegaspargase administration to the index event was 20 days in the non-SC group (first occurrence of the lowest fibrinogen or highest APTT) and 22 days in the SC group (first occurrence of grade 4 fibrinogen reduction or grade ≥3 APTT prolongation). Regarding temporal distribution, the cumulative incidence of SC increased over time, with rates of 25.84% (92/356) at 1 month, 28.65% (102/356) at 3 months, 32.87% (117/356) at 6 months, and 38.48% (137/356) at 1 year.

**TABLE 2:** Characteristics after pegaspargase exposure and variable assignments.

Code	Variable and assignments	Non-SC (*n* = 218)	SC (*n* = 138)	*P* value
X1	Regimen	​	​	0.600
	‘VDLP/VDLD’ = 1	185 (84.86)	122 (88.41)	​
	‘CAML’ = 2	30 (13.76)	14 (10.14)	​
	‘HR-1’/‘HR-2’/‘HR-3’ = 3	3 (1.38)	2 (1.45)	​
X2	Dose number	​	​	0.176
	‘≤2’ = 1	146 (66.97)	93 (67.39)	​
	‘>2, ≤4’ = 2	35 (16.06)	14 (10.14)	​
	‘>4’ = 3	37 (16.97)	31 (22.46)	​
X3	Albumin (g/L)	35.8 (32.6, 38.8)	34.2 (31.0, 36.7)	<0.001***
X4	ALT (U/L)	32 (21, 48)	36 (24, 59)	0.020*
X5	AST (U/L)	24 (19, 33)	25 (19, 35)	0.481
X6	TBIL (μmol/L)	12 (8, 16)	22 (19, 35)	<0.001***
X7	CRP (mg/L)	2.25 (2.18, 2.33)	2.08 (1.97, 2.19)	0.096
X8	PLT (×10^9^/L)	105 (61, 182)	88 (48, 135)	0.005**
X9	PCT (μg/L)	0.04 (0.04, 0.05)	0.09 (0.04, 0.22)	<0.001***
​	Severe coagulopathy	‘No’ = 0	‘Yes’ = 1	​

Abbreviations: VDLP/VDLD: VCR + DNR + PEG-ASP + P red/Dex; CAML: CTX + Ara-C+6-MP + PEG-ASP; VCR, vincristine; DNR, daunorubicin; PEG-ASP, PEG-asparaginase; Pred, prednisone; Dex, dexamethasone; CTX, cyclophosphamide; Ara-C, cytarabine; 6-MP, 6-mercaptopurine; ALT, alanine aminotransferase; AST, aspartate aminotransferase; TBIL, total bilirubin; CRP, C-reactive protein; PLT, platelets; PCT, procalcitonin; IQR, interquartile range.

All quantitative data are presented as median (IQR), and categorical data are expressed as n (%). Significance: *P < 0.05, **P < 0.01, ***P < 0.001.

### 3.2 Screening of independent risk factors for SC

Stepwise regression initially identified four potential risk factors: treatment regimen, albumin, total bilirubin, and PCT. These variables were subsequently included in a multivariate logistic regression analysis, revealing that low albumin, high total bilirubin, and elevated PCT are independent risk factors for SC (all *P* < 0.05).

### 3.3 Construction and validation of the nomogram prediction model

#### 3.3.1 Model construction

Based on the three independent risk factors identified by multivariate logistic regression, a nomogram for predicting SC risk was constructed, enabling clear quantification of an individual’s likelihood of developing SC (Figure 1A). The odds ratios (ORs) and 95% confidence intervals (CIs) for each independent risk factor are presented in Figure 1B.

**FIGURE 1:**
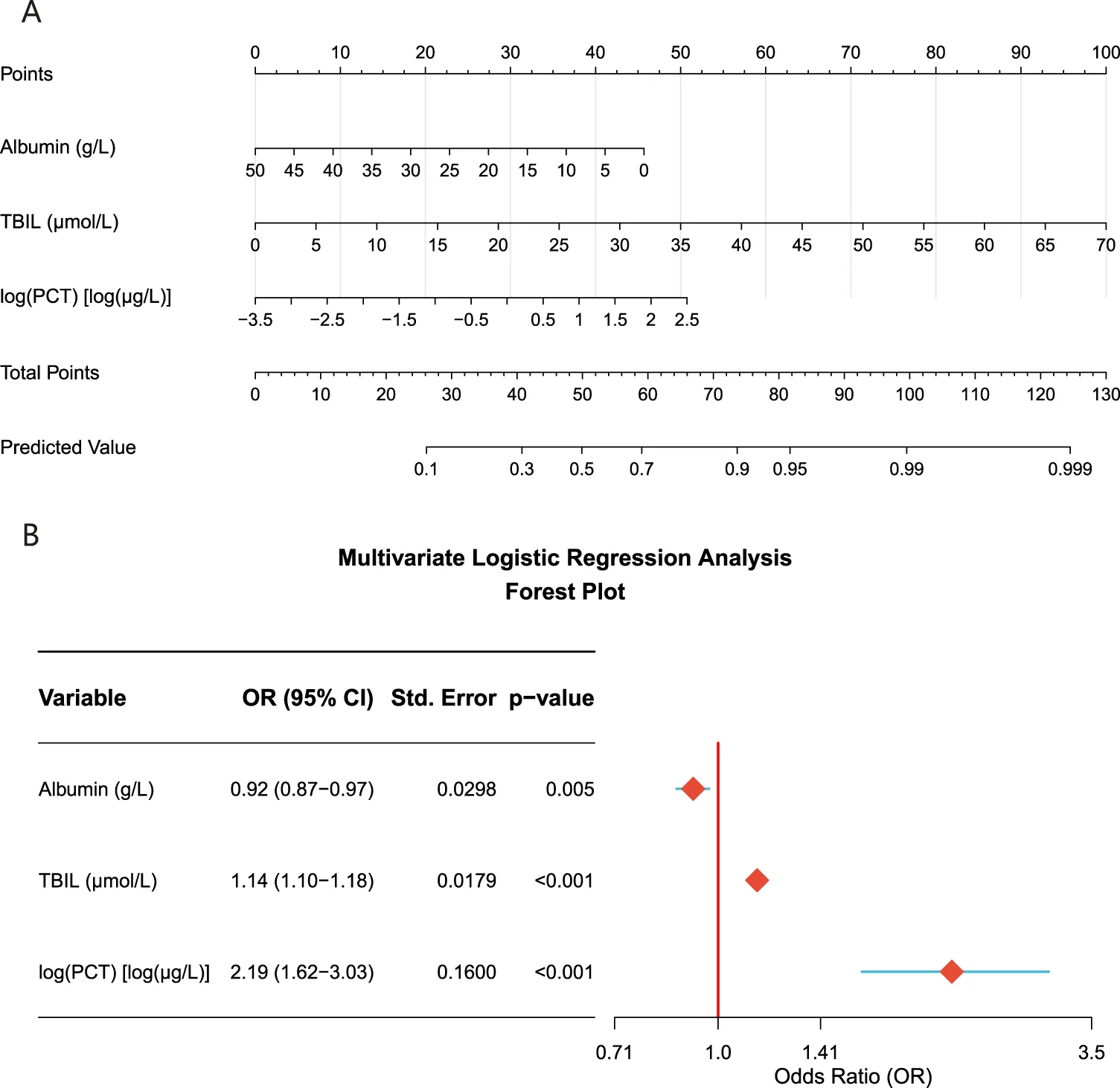
Prediction model for severe coagulopathy (SC) and its independent risk factors. **(A)** Nomogram for predicting the risk of SC. Three independent risk factors are integrated in the model: albumin level, total bilirubin level, and procalcitonin (PCT) level. The total score is calculated by summing the scores corresponding to each indicator to predict the probability of SC occurrence. **(B)** Forest plot of SC risk factors displaying the odds ratio (OR) and 95% confidence interval (CI) of each independent risk factor, clarifying the impact strength of each factor on SC occurrence. TBIL, total bilirubin; PCT, procalcitonin; log (PCT): log transformation of PCT.

#### 3.3.2 Website of nomogram

A web-based nomogram calculator for SC has been developed and is freely accessible online (https://286664940.github.io/coagulopathy/). This tool is designed to provide diagnostic probability, enabling healthcare professionals to identify SC risks at an early stage. By simply inputting patient values, the tool generates accurate, accessible, and visual predictions.

#### 3.3.3 Model discrimination and calibration

The ROC curve for this prediction model demonstrated an AUC of 0.819 (95% CI: 0.773–0.864), reflecting excellent discriminative performance (Figure 2A). The model thus showed a satisfactory ability to differentiate between patients who did and did not develop SC. The calibration curve showed a good fit between the nomogram-predicted SC risk and the observed incidence, with a Brier score of 0.160 (Figure 2C), indicating that the predicted probabilities were generally consistent with the actual event rates. Internal validation, conducted via bootstrap resampling (1,000 repetitions), yielded a corrected AUC of 0.814 (95% CI: 0.767–0.860; Figure 2B). The small difference between the apparent AUC (0.819) and the bootstrap-corrected AUC (0.814) suggested that overfitting was limited. Furthermore, the calibration curve from internal validation confirmed robust agreement between predicted and observed values (Figure 2D).

**FIGURE 2:**
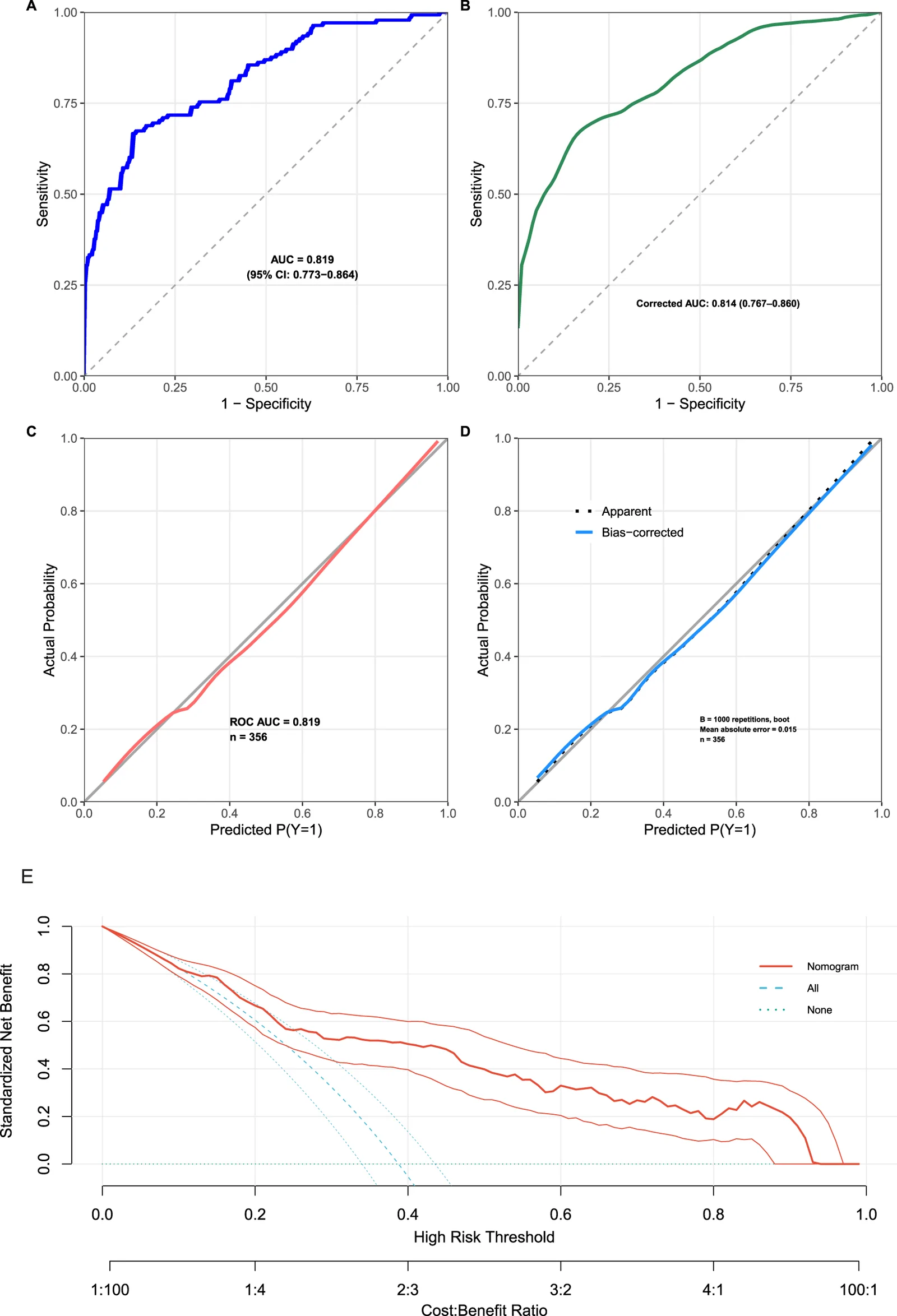
Receiver operating characteristic (ROC) and calibration curves of the prediction model and decision curve analysis. **(A)** ROC curve of the model (area under the curve [AUC] = 0.819); **(B)** ROC curve after 1,000 bootstrap validations (corrected AUC = 0.814); **(C)** calibration curve of the model; **(D)** calibration curve after validation; **(E)** Decision curve analysis (DCA): when the threshold probability is between 0 and 0.93, the model has a positive standardized net benefit.

#### 3.3.4 Model clinical applicability

DCA results revealed that the nomogram model yielded a positive standardized net benefit across thresholds from 0 to 0.93, indicating that, within this range, use of the model to guide clinical decision-making would provide a greater net benefit than either the “treat all” “treat none” strategy. Hence, these findings suggest the model’s potential clinical utility within this range (Figure 2E).

### 3.4 Multivariate analysis of blood product costs

Of the 356 patients analyzed, 283 underwent blood transfusions. Among these individuals, 67 received only plasma, 52 received only cryoprecipitate, and 164 received both blood products. The median cost of blood products was 2,143 renminbi (RMB), with costs reaching a maximum of 14,340 RMB.

To further elucidate the factors affecting blood product costs, potential variables such as patient age, weight, ALL risk stratification, blood product type, transfusion count, occurrence of SC, and year of initial ALL diagnosis were examined. Stepwise multiple linear regression identified patient age, blood product type, transfusion count, and year of initial ALL diagnosis as independent predictors of blood product costs (Figure 3). The overall regression model was significant (F = 115.972, *P* < 0.001), and the adjusted *R* ^2^ of 0.677 indicated that the included variables accounted for approximately 67.7% of the variance in blood product costs. The DW test statistic was 1.640, indicating independence of the residuals, whereas the VIF values ranged from 1.000 to 2.000, confirming the absence of multicollinearity.

**FIGURE 3:**
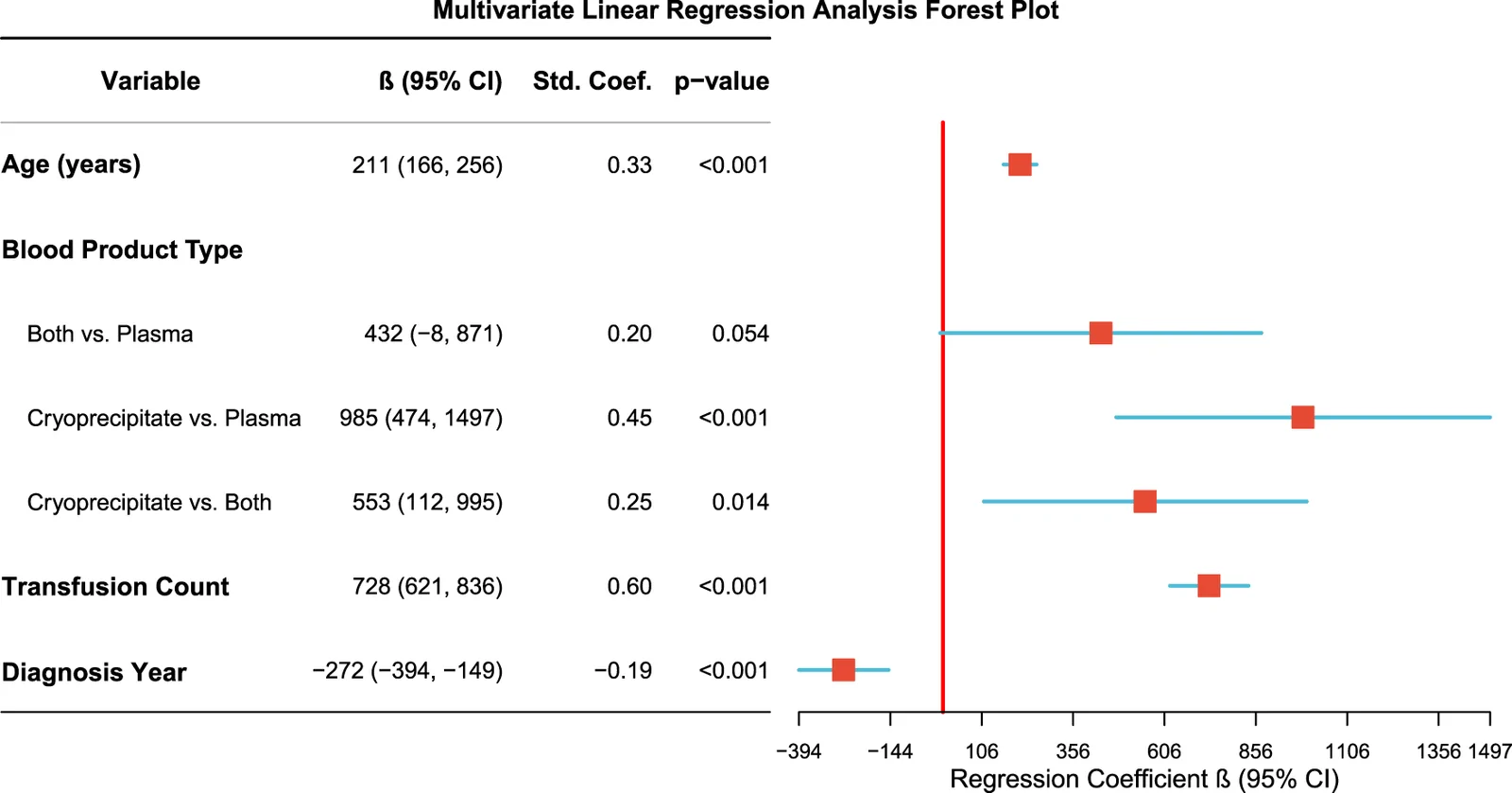
Independent factors associated with increased blood product costs. Estimated marginal increase in cost (with 95% confidence intervals [CIs]) is presented for significant independent variables. The analysis adjusted for relevant clinical covariates, and the adjusted *R* ^2^ value for the model is 0.677. Transfusion count denotes the number of pegaspargase-related transfusion events (plasma or cryoprecipitate administered for coagulopathy), with multiple transfusions within the same exposure counted as one event; costs were calculated based on actual transfusion costs.

## 4 Discussion

This study was conducted to evaluate the risk prediction of pegaspargase-associated SC and to quantify its health economic impact. Pegaspargase functions by depleting asparagine, thereby inhibiting hepatic protein synthesis and resulting in decreased levels of proteins including antithrombin III, protein C, protein S, fibrinogen, and plasminogen. This mechanism produces both prothrombotic and pro-hemorrhagic effects (Goyal and Bhatt, 2015; Ranta et al., 2013). This study focused specifically on laboratory-defined severe coagulopathy, which primarily manifests as severe hypofibrinogenemia induced by this mechanism. In these patients, coagulation screening tests often revealed abnormalities, including prolonged prothrombin time and APTT, alongside significantly reduced fibrinogen levels (Xu et al., 2016). Given this mechanism, the primary objective of the present study was to develop a predictive model for SC based on readily available clinical indicators.

The results of this study showed that among pediatric patients with ALL receiving the standard CCLG-ALL-2018 treatment, the overall incidence of pegaspargase-induced SC was 38.76% (138/356), with 87.68% (121/138) of cases presenting as marked reductions in fibrinogen. Reported incidences of pegaspargase-associated coagulation abnormalities vary widely owing to differences in administration regimens, observation periods, and definitions of coagulopathy across studies; nonetheless, all reports consistently describe significant fibrinogen reduction (Wang et al., 2018; Wang et al., 2020). Pegaspargase-induced coagulopathy typically initiates a decline in laboratory parameters 4–5 days after the first dose (Zhang et al., 2018), with peak changes occurring at approximately 11 days (Xu et al., 2016; Zhang et al., 2018). The current study revealed varying degrees of abnormalities in coagulation parameters within 5 days of pegaspargase administration and identified the high-risk window for SC at approximately 20–22 days. This discrepancy may reflect the broader chemotherapy cycle analyzed in this study compared with the induction-phase focus of earlier research. Nevertheless, the peak window for SC remained within 1 month, providing a critical reference point for optimizing clinical monitoring strategies. These observations underscore the clinical significance of coagulopathy as an adverse effect warranting close surveillance during pegaspargase therapy.

Coagulopathy occurred most frequently during the VDLP or VDLD phases of treatment. This finding aligns with existing reports indicating a high prevalence of pegaspargase-associated venous thromboembolic events during the glucocorticoid-inclusive induction phase (Cao et al., 2026; Rozen et al., 2017). However, the underlying mechanisms may differ. In the current study, coagulopathy primarily manifested as a reduction in fibrinogen, likely attributable to pronounced inhibition of hepatic synthetic function by pegaspargase during this phase. Conversely, literature attributes thrombotic events to the combined effect of decreased anticoagulant proteins and the procoagulant effect of glucocorticoids (Kłosowski et al., 2022). This temporal concurrence of these risks suggests that the VDLP/VDLD phase is a high-risk period for pegaspargase-associated coagulation system disturbances, which may simultaneously lead to a complex state of bleeding and thrombotic complications. Accordingly, clinical management necessitates dual monitoring and assessment strategies.

Notably, multivariate analysis identified decreased albumin, elevated total bilirubin, and increased PCT as independent risk factors for SC in this pediatric cohort. These factors are consistent with previously reported grade ≥3 adverse reactions to pegaspargase, including hypoalbuminemia, hepatotoxicity, and coagulopathy (Heo et al., 2019). Their coexistence reflects a shared pathophysiological axis involving liver injury, coagulation disorder, and systemic inflammation rather than isolated organ-specific insults. Specifically, hepatotoxicity, hypoalbuminemia, and coagulopathy represent different manifestations of pegaspargase-induced impairment of hepatic metabolism and protein synthesis (Pourhassan et al., 2023).

A decreased albumin level serves as a direct indicator of impaired hepatic synthetic function. Pegaspargase inhibits protein synthesis, reducing the production of coagulation factors, including fibrinogen (Ranta et al., 2013). Because albumin shares hepatocyte synthetic capacity with fibrinogen, antithrombin III, and other coagulation proteins, their plasma levels fall together once asparagine becomes limiting (Wang et al., 2020). Thus, hypoalbuminemia reflects both synthetic decline and a systemic hyperinflammatory state, linking nutritional status, inflammation, and coagulopathy (Başaran et al., 2023; Markanday, 2015).

Elevated total bilirubin indicates pegaspargase-associated hepatotoxicity, signifying hepatocyte damage or cholestasis. Given that the liver produces most coagulation factors, significant impairment of hepatic synthetic function reduces their production (Heo et al., 2019; Popa et al., 2026). Pegaspargase-related hepatotoxicity can manifest as both elevated bilirubin and impaired synthetic function, leading to concurrent reductions in fibrinogen, antithrombin III, and other coagulation proteins (Juluri et al., 2022).

PCT is a robust marker of systemic inflammatory response. In pediatric ALL, elevated PCT reflects not only bacterial infection but also noninfectious stress, such as tissue injury and ischemia–hypoxia (Popa et al., 2026). Inflammation promotes coagulopathy through endothelial injury, massive tissue factor release from inflammatory cells and platelets (Musgrave et al., 2023), and suppression of physiological anticoagulants, while enhancing Plasminogen activator inhibitor-1 (PAI-1) and Thrombin-activatable fibrinolysis inhibitor (TAFI) activity. These processes may contribute to increased thrombin generation and microvascular thrombosis, followed by consumption of coagulation factors and platelets and, in some circumstances, progression toward hypocoagulability and bleeding (Iba et al., 2021).

Thus, decreased albumin, elevated total bilirubin, and increased PCT levels collectively constitute a risk framework for pegaspargase-associated SC, viewed from three interrelated pathophysiological perspectives: hepatic synthetic inhibition with inflammatory load, hepatic parenchymal injury, and systemic inflammatory activation. This provides an essential mechanistic foundation and clinical reference for the early identification of children at high risk and for tailored monitoring and intervention. Although derived from a single-center cohort, the predictors identified in this model reflect fundamental pathophysiological processes—hepatic synthetic impairment, hepatotoxicity, and systemic inflammation—that are not unique to our institution. Therefore, while the exact coefficients and cutoff values may require recalibration in other centers, the overall modeling framework and selected variables are expected to retain predictive utility across diverse clinical environments.

Both domestic and international guidelines recommend blood product support for pegaspargase-associated coagulation abnormalities. When fibrinogen is <1.0 g/L, close monitoring and corrective measures, including blood product support, are advised (Chinese Medical Association of Emergency Medicine; Chinese Medical Doctors Association of Interventional Doctors; Hemorrhage Professional Committee of Chinese Research Hospital Association; Chinese Federation of Hemorrhage Centers, 2023; Expert Working Group on Perioperative Intervention, Interventional Physician Branch of Chinese Physician Association, and Chinese Venous Interventional Alliance, 2025). Asparaginase-induced coagulopathy is biphasic; if significant bleeding occurs and coagulopathy is confirmed, appropriate blood product replacement can be administered, though excessive supplementation may increase the risk of thrombosis (National Health Commission of the People’s Republic of China, 2018). In cases of bleeding, fresh frozen plasma should be avoided because its asparagine content may counteract pegaspargase activity. If fibrinogen is <0.5 g/L and correction is required, conservative management with cryoprecipitate is preferred (Beavers et al., 2022).

In the current study, the impact of pegaspargase-associated coagulopathy on blood product costs was systematically analyzed in a pediatric population. Multivariate analysis identified patient age, blood product type, transfusion count, and year of diagnosis as independent factors affecting blood product costs. Among these variables, the type and number of blood products were the primary determinants. Differences in the unit price of plasma and cryoprecipitate, as well as the selection and combination of products, contributed to differences in the overall costs, while additional transfusions further increase expenses.

The finding that age—rather than body weight—was an independent factor reflects the high collinearity between these variables. In pediatric populations, age, as a composite variable, encompasses physical information attributed to weight and correlates with other clinical characteristics. In clinical practice, transfusion decisions are individualized, based on the severity of coagulopathy and bleeding risk, both of which are more often associated with the patient’s age than weight-based dosing alone. The effect of the year of diagnosis likely reflects changes in medical insurance reimbursement policies and clinical transfusion strategies. Specifically, our center has observed a trend toward stricter intervention thresholds for coagulation dysfunction and transfusion timing in recent years. For patients who do not achieve a grade 4 fibrinogen reduction, clinicians increasingly use plasma rather than cryoprecipitate. Although cryoprecipitate is effective for correcting hypofibrinogenemia (Ayaganov et al., 2024), this strategic shift may reflect a more conservative balance between correcting coagulopathy and minimizing the risk of thrombosis. Clinicians should focus on the comprehensive assessment of liver injury and mild suppression of multiple coagulation factors, rather than isolated parameter changes. Additional considerations include the stability of cryoprecipitate and specific adjustments to insurance reimbursement policies, which may further affect clinical strategies.

This study has several limitations. First, as a single-center retrospective study, it is inherently susceptible to selection and information biases. Our findings may not fully reflect the demographic, laboratory, or therapeutic heterogeneity of other institutions. Second, the use of stepwise regression for variable selection carries a recognized risk of overfitting and predictor instability, particularly in a relatively limited sample. Although internal validation was performed using bootstrap resampling, external validation in an independent multicenter cohort is still lacking. Third, the cost analysis was restricted to direct blood product costs and did not account for additional expenses related to prolonged hospitalization, enhanced monitoring, or treatment adjustments, potentially underestimating the overall economic burden. Fourth, discrepancies exist between laboratory abnormalities and clinically apparent coagulopathy; biochemical changes do not invariably correspond to meaningful clinical events and may manifest differently across individuals. In light of these limitations, the current model should be interpreted as a hypothesis-generating tool to support biochemical risk stratification rather than as a definitive clinical decision-making instrument. External validation in independent multicenter cohorts is warranted, and, where necessary, model recalibration should be performed before broader implementation.

In summary, this study successfully developed and internally validated a nomogram model incorporating albumin, total bilirubin, and PCT levels to effectively predict the risk of pegaspargase-associated SC in children with ALL. The model demonstrated promising discriminative performance and clinical utility within the study population. Furthermore, findings indicate that coagulopathy management significantly increases blood product-related expenses and highlight critical influencing factors. These observations suggest that early prediction and individualized intervention could enhance treatment safety and yield health and economic benefits, offering valuable insights for achieving precise, efficient, and economical treatment management of pediatric ALL.

## Data Availability

The raw data supporting the conclusions of this article will be made available by the authors, without undue reservation.
